# Obsessive Beliefs, Metacognitive Beliefs, and Rumination in Parents of Adolescents with and Without Obsessive–Compulsive Disorder: A Linear Mixed-Effects Model

**DOI:** 10.3390/brainsci15101093

**Published:** 2025-10-10

**Authors:** Emre Mısır, Mutlu Muhammed Özbek

**Affiliations:** 1 Department of Psychiatry, Faculty of Medicine, Baskent University Ankara Hospital, Ankara 06490, Turkey; 2 Department of Interdisciplinary Neuroscience, Graduate School of Health Sciences, Ankara University, Ankara 06340, Turkey; 3 Department of Child and Adolescent Psychiatry and Mental Health, Faculty of Medicine, Yalova University, Yalova 77200, Turkey; mutlu.ozbek@yalova.edu.tr

**Keywords:** obsessive–compulsive disorder, obsessive beliefs, metacognition, rumination

## Abstract

Background: Parental cognitive characteristics may represent environmental risk factors in obsessive–compulsive disorder (OCD). This study compared obsessive beliefs, metacognitions, and ruminative thinking in parents of adolescents with OCD and healthy controls (HCs), and examined links with clinical features in patients. Methods: Participants were 45 adolescents with OCD, 45 HCs, and both their mothers and fathers. The Children’s Yale-Brown Obsessive Compulsive Scale (CY-BOCS) assessed symptom severity in adolescents. Parents completed the Obsessive Beliefs Questionnaire (OBQ), Ruminative Thought Style Questionnaire (RTSQ), 30-item Metacognitions Questionnaire (MCQ-30), and Patient Health Questionnaire-9 (PHQ-9). Data were analyzed using linear mixed-effects models, followed by correlation and regression analyses. Results: Parents of patients had higher scores on the importance/control of thoughts, the need to control thoughts, and cognitive self-consciousness (MCQ-CSC). Mothers of adolescents with OCD had the highest scores on inflated responsibility/threat estimation (OBQ-RTE), perfectionism/intolerance of uncertainty (OBQ-PIU), rumination, and cognitive confidence (MCQ-CC). Regression analyses showed that lower maternal MCQ-CC predicted earlier OCD onset, while higher rumination predicted later onset. Obsession severity in adolescents was linked to higher maternal MCQ-CSC, obsessive slowness to maternal OBQ-PIU, and pathological doubt to greater maternal rumination. Children’s indecisiveness correlated with paternal OBQ-RTE and OBQ-PIU. Conclusions: Our findings revealed elevated cognitive vulnerabilities for OCD in mothers of affected adolescents and identified specific associations between parental cognitive characteristics and their children’s symptom profiles. Future longitudinal studies using dyadic parental design with larger samples may further elucidate the role of parental cognitive patterns in the development and course of OCD.

## 1. Introduction

Obsessive–compulsive disorder (OCD) is a neuropsychiatric condition characterized by obsessions and compulsions [1]. Early-onset OCD differs from adult-onset OCD in terms of clinical features and heritability, with stronger links to genetic vulnerability [2,3,4]. Nevertheless, environmental and parental factors may also play an important role in early-onset OCD, because this stage represents a sensitive period of development with heightened susceptibility to environmental inputs [5,6,7]. Given that the impact of environmental factors tends to diminish with age, early identification and intervention of risk factors in childhood is of great importance [8,9].

The primary strategy for identifying targets of early intervention is to determine the factors that increase vulnerability to OCD. Cognitive characteristics linked to clinical symptoms, subclinical manifestations in the general population, or elevated risk of developing OCD over time are referred to as cognitive vulnerability factors [10,11,12]. According to cognitive-behavioral models, core domains of such vulnerability include obsessive beliefs, particularly inflated responsibility and threat overestimation, perfectionism and intolerance of uncertainty, and beliefs about the importance and control of thoughts [13,14,15]. In addition to obsessive beliefs, metacognition is considered a key factor in the development of OCD [16,17]. The metacognitive model posits that individuals’ knowledge of their cognitive processes and their regulation strategies play a critical role in both the onset and maintenance of the disorder [18]. Specifically, beliefs that thoughts are uncontrollable or dangerous, reduced confidence in memory, and the urge to control thoughts due to fear of harm contribute to the persistence of obsessions [19,20,21]. Thus, obsessive beliefs and metacognitive dysfunctions represent important cognitive risk factors for OCD. Notably, elevated levels of these vulnerabilities have also been observed in relatives of individuals with the disorder [5,6,22].

Parental cognitive vulnerabilities are crucial for understanding early-onset OCD, as parents’ thinking styles constitute environmental factors that may shape the development of pediatric OCD [23,24]. Based on genetic predispositions, parents’ cognitive tendencies can foster cognitive vulnerabilities in children through learning. Indeed, several studies have reported significant correlations between children’s and parents’ attribution styles and cognitive schemas [25,26,27]. Research on learning theory in child psychopathology has mainly focused on anxiety disorders, showing that parents’ negative thinking patterns can be transmitted to their children [28,29,30]. Nevertheless, studies specifically examining the relationship between parental thinking styles and OCD symptoms in children remain limited.

Relatives of patients with OCD are at increased risk for obsessive-compulsive symptoms at both clinical and subclinical levels [31,32,33,34]. Moreover, exaggerated threat estimation, inflated responsibility, perfectionism, and intolerance of uncertainty have been found to be higher among OCD relatives [5,35]. A study conducted in the general population demonstrated that adolescents’ inflated responsibility and threat estimation were associated with parental OCD symptom severity [36]. Furthermore, associations have been demonstrated between metacognitive beliefs and the severity of obsessive-compulsive symptoms in both patients diagnosed with OCD and healthy individuals [37,38,39]. These findings suggest that cognitive vulnerability factors contribute to susceptibility to OCD symptoms. Therefore, investigating the relationships between parents’ cognitive vulnerabilities and the clinical symptoms of OCD patients could help to elucidate the etiopathogenesis of the disorder. Indeed, cognitive characteristics transmitted through learning may trigger symptom development in children. Although few studies have examined the relationship between parents’ obsessive beliefs and symptoms of children, no study has investigated the relationship between parents’ metacognitive beliefs and clinical symptoms in children with OCD. One study reported a positive association between mothers’ responsibility and threat estimation (RTE) and importance and control of thoughts (ICT) and the severity of obsessive-compulsive symptoms in children, whereas another found no such relationship [6,40]. Studies on obsessive beliefs in parents of early-onset OCD patients have primarily focused on mothers or primary caregivers [6,40]. However, maternal and paternal influences may differ, as studies evaluating parental OCD diagnoses or symptom severity have found differences between mothers and fathers [41,42,43]. These findings suggest that different parental roles may have distinct effects on child psychopathology. Yet, existing results are inconsistent, and no study has simultaneously examined cognitive vulnerability traits in both parents in relation to their children’s clinical characteristics. Therefore, assessing both mothers and fathers may provide more specific insights. To our knowledge, this study is the first to compare the thinking styles of mothers and fathers of children and adolescents diagnosed with OCD.

Similarly to obsessions, rumination—a repetitive thought pattern—is a common cognitive process in OCD. It involves persistent thoughts about a specific topic in response to uncertainty, goal-situation mismatches, or negative emotions [44]. Unlike obsessions, rumination does not necessarily accompany compulsions and can occur throughout the day on various topics. It is also associated with neurocognitive processes such as inhibition and set-shifting [45,46,47]. Although obsessions and ruminations share some features, ruminations are typically verbal, past-oriented, and difficult to control, whereas obsessions can also involve images or urges, are often experienced as ego-dystonic, and are usually suppressed [48,49,50]. Rumination frequency is higher in individuals with OCD than in the general population, and it correlates with obsessions independently of depression [51,52]. A recent machine learning study identified rumination as a transdiagnostic factor most strongly associated with disorders including depression, bipolar disorder, anxiety disorders, and OCD [53]. These findings suggest that a tendency toward ruminative thinking may be a symptom of OCD and a marker of susceptibility. However, the relationship between ruminative thinking tendencies in parents and obsessive-compulsive symptoms in children and adolescents has yet to be investigated.

The main objective of this study is to compare the cognitive vulnerability components of OCD, such as obsessive beliefs, metacognition and rumination between parents of children diagnosed with OCD and parents of children without OCD. Our first hypothesis is that parents of children with OCD exhibit higher levels of obsessive beliefs, metacognitive dimensions, and rumination. Additionally, we hypothesize that the difference in ruminative thinking tendencies remains significant even when controlling for parents’ depressive symptom levels. To test this hypothesis, it may be useful to divide the disorder into more homogeneous groups to identify more specific relationships. Indeed, different mechanisms may be at play in the development of autogenous and reactive obsessions. Studies investigating metacognitive differences among types of obsessions have yielded conflicting results [54,55]. According to the cognitive-behavioral perspective, autogenous obsessions are linked to beliefs about the importance and control of thoughts, while reactive obsessions are associated with beliefs about responsibility [56]. Based on this information, our second hypothesis is that the importance and control of thoughts are higher in parents of adolescents with autogenous obsessions. Our third hypothesis is that the levels of obsessive beliefs, metacognition, and rumination in parents are positively associated with the clinical scores of their children.

## 2. Materials and Methods

### 2.1. Participants

The study sample consisted of an adolescent group (ages 10–17), including patients diagnosed with OCD according to the Diagnostic and Statistical Manual of Mental Disorders, Fifth Edition (DSM-5) criteria, as well as age- and gender-matched healthy controls. (HCs). There was also a family group composed of the parents (both mothers and fathers) of the adolescents. Data were collected between October 2022 and September 2023. Written informed consent was obtained from all participants, as well as from the parents of the adolescent participants.

Patients were consecutively included in the study following a detailed psychiatric evaluation conducted by one of the researchers, an experienced child and adolescent psychiatrist (MMÖ). These evaluations were conducted among individuals presenting to the child and adolescent psychiatry and mental health outpatient clinic of Kars State Hospital, in Kars Province, located in northeastern Turkey. Healthy controls (HCs) were also included and recruited through a convenience sampling method, including children of hospital staff and potential candidates identified through the authors’ personal networks. The same researcher (MMÖ) obtained informed consent from the adolescents and their parents and administered the Structured Clinical Interview for DSM-5 Disorders—Clinician Version (SCID-5-CV) to assess the parents’ mental health. Based on the inclusion and exclusion criteria, data were initially collected from 53 patients and 57 HCs. However, due to incomplete or improperly completed questionnaires from the parents of eight patients and 12 HCs, the analyses included data from 45 patients (26 females, average age = 13.76 ± 2.02 years), 45 HCs (25 females, average age = 13.29 ± 2.21 years), and 90 parents.

The inclusion criteria for patients and HCs were being between 10 and 17 years of age. The exclusion criterion for HCs was having any past or current psychiatric diagnosis. Patients were excluded if their OCD onset was secondary to an organic condition, such as pediatric acute-onset neuropsychiatric syndrome (PANDAS), or to acute psychogenic causes, such as traumatic events. Patients with a comorbid diagnosis of substance use disorder, psychotic disorder, bipolar disorder, or intellectual disability were also excluded. For both patients and HCs, the presence of any condition that could affect cognitive functioning, such as epilepsy or neurometabolic diseases, was an exclusion criterion. Participants were also excluded if either parent had a diagnosis of a substance use disorder, schizophrenia, bipolar disorder, an intellectual disability, or a neurodegenerative disease that causes cognitive impairment.

The study was approved by the Clinical Research Ethics Committee of Kars State Hospital (date: 26 October 2021 and number: 80576354-050-99/262). It was conducted according to the principles of the Helsinki Declaration, and written informed consent was obtained from each participant.

### 2.2. Instruments

The Children’s Yale-Brown Obsessive Compulsive Scale (CY-BOCS) was administered by the researcher MMÖ to assess obsessive-compulsive symptoms in patients. Self-report measures were used to evaluate obsessive beliefs, ruminative thinking style, metacognitions, and the severity of depressive symptoms, respectively: the Obsessive Beliefs Questionnaire (OBQ), the Ruminative Thought Style Questionnaire (RTS-Q), the 30-item Metacognitions Questionnaire (MCQ-30), and the Patient Health Questionnaire-9 (PHQ-9) for parents.

Autogenous obsessions tend to arise abruptly, often without any identifiable external triggers [57]. Among the participants, dominant obsessions such as contamination were classified as reactive, whereas aggression, sexual, magical/superstitious, somatic, and religious obsessions were classified as autogenous [58].

#### 2.2.1. Children’s Yale-Brown Obsessive Compulsive Scale

The CY-BOCS is a semi-structured interview used to assess the presence and severity of obsessive-compulsive symptoms in individuals aged 6–17. It consists of two main parts: a severity scale scored by the clinician and a symptom checklist [59]. The severity scale includes 18 items that assess obsessions, compulsions, level of insight, avoidance, indecisiveness, excessive responsibility, obsessive slowness, pathological doubt, and global severity and improvement. Scoring of the scale is based on the first ten items, yielding a total score of 0–40. The Turkish version of the CY-BOCS scale demonstrated an internal consistency of 0.77, and the intraclass correlation coefficients for the compulsion and obsession subscales and the CY-BOCS total score were 0.85, 0.94, and 0.89, respectively [60].

#### 2.2.2. Obsessive Beliefs Questionnaire

The OBQ is a 44-item self-report scale that assesses obsessive beliefs. It consists of three subscales: Responsibility/Threat Estimation (OBQ-RTE), Perfectionism/Intolerance of Uncertainty (OBQ-PIU), and Importance of Thoughts/Control of Thoughts (OBQ-ICT). The corrected item-total correlations ranged from 0.35 to 0.64, and the internal consistency coefficients for the subscales ranged between 0.86 and 0.88 [61]. The three-factor structure of the Turkish version has been validated. The three-factor structured model produced a Root Mean Square Error of Approximation (RMSEA) of 0.08, a Comparative Fit Index (CFI) of 0.94, and a Standardized Root Mean Square Residual (SRMR) of 0.08 [61].

#### 2.2.3. Ruminative Thought Style Questionnaire

The RTS-Q is a 7-point Likert-type self-report scale designed to assess an individual’s tendency toward ruminative thinking, independent of the current mood state [62]. This feature suggests that the scale may also be useful in other psychopathological conditions. In the Turkish version of the scale, the single factor was found to explain 63.43% of the total variance. The internal consistency coefficient of the single-factor structure of the Turkish version was found to be 0.91, and the test–retest reliability coefficient was 0.84 [63].

#### 2.2.4. Metacognitions Questionnaire

The MCQ-30, developed by Cartwright-Hatton and Wells, is a 30-item, 4-point Likert-type self-report scale [64]. It consists of five subscales: Positive Beliefs About Worry (MCQ-PW), Negative Beliefs About Worry (MCQ-NW), Cognitive Confidence (MCQ-CC), Need to Control Thoughts (MCQ-NCT), and Cognitive Self-Consciousness (MCQ-CSC). The MCQ-PW subscale includes items reflecting the belief that worrying is useful for planning and problem-solving. The MCQ-NW subscale includes items related to the belief that worries are uncontrollable and that worry must be controlled to maintain functionality and safety. The MCQ-CC subscale assesses the individual’s lack of confidence in their attention and memory. The MCQ-NCT subscale consists of items concerning the need to control thoughts due to negative beliefs related to responsibility, punishment, and superstition. The MCQ-CSC subscale indicates a continuous preoccupation with one’s own thinking process. The structural validity of the 5-factor model of the Turkish version of the scale was demonstrated [χ^2^/df = 1.51 (*p* < 0.001), RMSEA = 0.051, CFI = 0.90, Goodness of Fit Index (GFI) = 0.90, Root Mean Square Residual (RMR) = 0.50]. Test–retest correlation coefficients ranged from 0.70 to 0.85 for the subscales, while the Cronbach’s alpha coefficient was 0.86 [65].

#### 2.2.5. Patient Health Questionnaire-9

The PHQ-9 is a self-report questionnaire designed to assess the severity of depressive symptoms. The scale includes 9 items that measure how frequently symptoms have been experienced over the past two weeks, with each item rated on a 4-point scale from 0 to 3. A diagnosis of major depressive disorder is suggested when at least one of the first two items and a total of five or more items receive a “positive” response (defined as a score of 2 or 3 on items 1–8, and greater than 1 on item 9). In this study, depression was diagnosed by clinical interview and the PHQ-9 was used to assess overall symptom severity. The Turkish version of the scale has a sensitivity of 76.0% and a specificity of 85.3% [66]. Due to the lack of previously reported validity and reliability values for the scale, we conducted an exploratory factor analysis to assess validity and calculated Cronbach’s alpha to assess reliability. The PHQ-9 single-factor model was appropriate (Kaiser-Meyer-Olkin = 0.84), accounting for 43.4% of the total variance. Factor loadings ranging from 0.47 to 0.84 revealed that all items in the scale had adequate loadings for the factor. Internal consistency was high (Cronbach’s alpha = 0.88). The results indicated sufficient validity and reliability of the scale according to the criteria suggested in the literature [67].

### 2.3. Statistical Analysis

Statistical analyses were performed using R version 4.1.1 (The R Project for Statistical Computing). A power analysis was conducted to determine the sample size required to detect the interaction between adolescent diagnostic group (patient vs. control) and parent type (mother vs. father) with three predictors: diagnostic group, parent type, and their interaction (WebPower package version 0.9.4) [68]. Assuming a medium effect size (Cohen’s f^2^ = 0.15), α = 0.05, and power = 0.80, a total of 77 observations (approximately 39 families) was required.

Participants with more than 10% missing data on any scale were excluded from the analyses (eight patient parents and 12 healthy control parent participants). Initially, missing values for the remaining participants were imputed using their mean score on the corresponding subscale. Then, the normality of the variables’ distributions was assessed based on their skewness and kurtosis values [69]. Although all scale scores showed normal or near-normal distributions, a logarithmic transformation was applied to improve model assumptions and estimation accuracy. Independent samples *t*-tests were conducted to compare continuous variables and chi-squared tests were conducted for categorical variables between the two groups. Logistic regression analysis using the enter method examined the likelihood of an OCD diagnosis among parents with parent type (mother or father) and adolescent diagnostic group factor (OCD or HCs) as predictor variables.

Linear mixed-effects (LME) models were used to assess within- and between-group differences in scale scores, accounting for the individual variability of the adolescent participants (lme4 package). LME models account for the non-independence of observations by including a random intercept for each family, addressing the correlation between both parents of an adolescent. This approach models within-family correlations, reducing the risk of inflated Type I error rates [70]. Additionally, LME offers a robust framework for analyzing the hierarchical data structure (parents nested within families) and is well-suited for the study’s sample size, providing more reliable estimates than traditional linear regression, which assumes independence of observations.

Separate models were constructed for each scale score. First, we examined interactions between the adolescent diagnostic group (OCD/HCs) and parent type (mother/father), and we included random effects for adolescent participants (see Model 1a). For the RTS-Q model, PHQ-9 scores were included as a fixed effect to control for depressive symptom severity (Model 1b). Because we aim to focus on dimensions of obsessive beliefs and metacognitions and total scores are unlikely to yield specific results, total MCQ-30 and OBQ scores were excluded from group comparisons.

Model 1a. Parent scale score ~ Adolescent group*Parent Type + (1| Adolescent ID)

Model 1b. Parent RTS-Q score ~ Adolescent group*Parent Type + PHQ-9 + (1| Adolescent ID)

Second, additional models were constructed for all scale scores, including the interaction between adolescent diagnostic group factor and obsession type (autogenous versus reactive). Adolescent participants were included as a random variable in these models. Degrees of freedom and *p*-values were calculated using Satterthwaite’s approximation. To control for multiple comparisons, the Benjamini–Hochberg procedure was applied to manage the false discovery rate (FDR). This approach was chosen over more conservative methods because it provides a better balance between controlling for false positives and maintaining statistical sensitivity. [71]. This improves the ability to detect meaningful effects and allows for a more specific exploration of relationships in subsequent analyses. Post hoc comparisons were performed using Tukey’s HSD test (emmeans package version 1.11.2-8).

Pearson correlation coefficients were calculated between patients’ clinical symptom scores and scale scores, showing either a significant main effect of adolescent group or a significant parent type × adolescent group interaction. Subsequently, backward linear regression analyses were performed using the patients’ gender and clinical symptom scores with multiple significant correlations as the dependent variables. Separate regression models were constructed for mothers’ and fathers’ scale scores. All analyses were two-tailed, and data are presented as mean ± SD unless otherwise specified.

## 3. Results

### 3.1. Sociodemographic and Clinical Characteristics

The patient and HC groups were similar in terms of age [t(88) = 1.04, *p* = 0.298] and gender [χ^2^(1) = 0.05, *p* = 0.832]. None of the adolescent participants reported a history of psychological trauma, suicide attempts, or self-harm behavior. The dominant obsession was reactive in 44.4% of the patients (*n* = 20) and autogenous in 55.6% (*n* = 25). The clinical characteristics of the patients are presented in Table 1.

Weak to very strong positive correlations (r = 0.241–0.989, all *p* < 0.05) were found between all scale scores of patients. Negative correlations were found between age of onset and CY-BOCS total score, as well as between age of onset and the obsession and compulsion subscales. The correlation matrix between clinical variables is shown in Table S1.

### 3.2. Comparison of Parents of Patients and Healthy Controls

Fathers [estimate(SE) = 3.178 (0.556), t = 5.707, *p* < 0.001] and parents of adolescents with OCD [estimate(SE) = 5.08 (1.299, t = 3.915, *p* < 0.001] had significantly higher mean age. Among the parents, 12 were diagnosed with OCD, 8 with major depressive disorder and 2 with generalized anxiety disorder (See Table 2). One parent of an adolescent with OCD had a history of suicide attempt.

Logistic regression analysis revealed a significant difference in the likelihood of OCD diagnosis across parental groups [Nagelkerke R^2^ = 0.231, χ^2^(2) = 16.852, *p* < 0.001]. The likelihood of having an OCD diagnosis was significantly higher among mothers (β= 2.579, Wald = 5.894, *p* = 0.015, OR = 13.19) and among parents of children diagnosed with OCD (β = 1.781, Wald = 4.927, *p* = 0.026, OR = 5.94). When groups were analyzed separately, in the parents of patients, OCD was more frequently diagnosed in mothers than in fathers [χ^2^(1) = 11.25, *p* < 0.001].

No alcohol or substance use disorders were identified in parents. Fathers (β = 0.649, Wald = 4.457, *p* = 0.035, OR = 1.91) and parents of children with OCD (β = 0.738, Wald = 5.762, *p* = 0.016, OR = 2.092) had a significantly higher likelihood of smoking.

In terms of the OBQ-ICT, MCQ-NCT, and MCQ-CSC scores, only the main effect of the adolescent diagnostic group was significant (see Table 3). However, both the main effect of the adolescent group and the adolescent group x parent type interaction had significant effects on the OBQ-RTE, OBQ-PIU, RTS-Q, and MCQ-CC scores. Post hoc analyses revealed that OBQ-RTE, OBQ-PIU, RTS-Q, and MCQ-CC scores were higher in OCD mothers than in their partners and higher in OCD mothers than in mothers of healthy adolescents. Additionally, the MCQ-CC score was higher in fathers of healthy adolescents than in their mothers. MCQ-PW and PHQ-9 scores were higher in mothers of patients than in their fathers but did not differ significantly between parents in terms of adolescent groups.

In the LME analyses evaluating the interaction between patient group (autogenous vs. reactive) and parent type on scale scores, mothers showed higher RTS-Q scores compared to fathers [estimate = −0.094 (SE = 0.028), t = −3.416, *p* = 0.021, ηp^2^ = 0.60]. However, no other significant main or interaction effects were found (see Table S2).

### 3.3. Relationships Between Clinical Scores of Patients and Scale Scores of Their Mothers

Correlation analyses revealed a significant, weak-to-moderate positive correlation between OBQ-RTE and pathological doubting. OBQ-PIU was positively associated with obsession severity and obsessive slowness (see Table S3). The RTS-Q showed significant positive correlations with age of onset and pathological doubting, while the MCQ-PW was positively correlated with pathological doubt. MCQ-NW was positively associated with avoidance and pathological doubt. A positive correlation was also observed between MCQ-CC and age of onset. Meanwhile, significant positive correlations were found between MCQ-CSC and CY-BOCS total score, as well as the compulsion, obsession, indecisiveness, and obsessive slowness subscales. On the other hand, a negative correlation was found between the MCQ-CSC and poor insight.

In linear regression analyses, age of onset was positively predicted by RTS-Q and negatively predicted by MCQ-CC [adjusted R^2^ = 0.238, F(2, 42) = 6.559, *p* = 0.003]. Meanwhile, the MCQ-CSC positively predicted obsession severity [adjusted R^2^ = 0.235, F(1, 43) = 13.239, *p* < 0.001], and the OBQ-PIU was associated with obsessive slowness [adjusted R^2^ = 0.156, F(1, 43) = 7.976, *p* = 0.007]. Additionally, RTS-Q scores positively predicted pathological doubt [adjusted R^2^ = 0.166, F(1, 43) = 8.567, *p* = 0.005]. The final models of the regression analyses are presented in Table 4.

### 3.4. Relationships Between Clinical Scores of Patients and Scale Scores of Their Fathers

A different pattern was present when correlations with the fathers’ scale scores were evaluated. Weak positive correlations were observed between excessive responsibility, indecisiveness, slowness, and OBQ-RT scores (see Table S4). Notably, indecisiveness showed significant correlations with both OBQ-RTE and OBQ-PIU. However, regression analysis was not performed due to the extremely high correlation between OBQ-RTE and OBQ-PIU scores in fathers of patients (r = 0.858, *p* < 0.001). Significant positive correlations were also found between doubt and both MCQ-CC and MCQ-NCT. In the regression analysis [adjusted R^2^ = 0.231, F(1, 42) = 10.83, *p* = 0.002], MCQ-NCT was associated with increased pathological doubt [β = 0.509, 95% CI (0.197, 0.821), t = 3.291, *p* = 0.002].

## 4. Discussion

In this study, we examined potential cognitive vulnerability factors for OCD among parents of adolescents. We found that all metacognitive beliefs, rumination, and obsessive beliefs—except for positive beliefs about worry—were elevated in parents of patients. Our primary focus was to determine whether these differences varied between mothers and fathers. Results indicated that, compared to fathers, mothers of patients had higher scores on measures of obsessive beliefs, rumination, and metacognitive beliefs. Furthermore, mothers and fathers exhibited different patterns of correlation between their children’s clinical measures and parental scale scores, suggesting distinct cognitive vulnerability profiles in mothers and fathers.

Numerous studies indicate that obsessive thoughts are fundamental to OCD development and that parental obsessive beliefs may contribute to early-onset OCD [5,22,56,72,73,74,75,76,77,78]. Nevertheless, studies on this topic are limited, and no prior research has compared obsessive beliefs in relatives of children and adolescents with OCD to those of healthy relatives. Examining relatives of healthy participants alongside proband relatives is an established approach for assessing parental cognitive characteristics as environmental risk factors [32,79]. To our knowledge, our study is the first to evaluate obsessive cognitions, rumination, and metacognitions from this perspective.

### 4.1. Differences Between Parents of Patients and Healthy Controls

A pioneering study by Rector et al. (2009) reported higher exaggerated threat estimation and inflated responsibility in adult relatives of OCD patients [5]. The same study also found that early-onset OCD subjects exhibited elevated levels of perfectionism and intolerance of uncertainty, along with exaggerated threat estimation and inflated responsibility, compared to healthy controls. Another study reported associations between maternal responsibility and thought suppression and the severity of OCD in children [8]. Consistent with these findings and the first hypothesis of our study, parents of adolescents with OCD showed increases across all subscales of the OBQ. Likewise, negative beliefs about worry, cognitive confidence, the need to control thoughts, and cognitive self-consciousness were elevated in parents of probands. Rumination scores were also higher in relatives of patients, independent of depressive symptom severity. Given that metacognitive beliefs and ruminative thinking, like obsessive beliefs, influence how events are interpreted and managed, these factors may have practical implications for communication and caregiving with children. To the best of our knowledge, our study is the first to evaluate the ruminative thinking tendency of parents of children with OCD.

One of the main objectives of our study was to investigate whether factors contributing to sensitivity to obsessions differed between mothers and fathers. Our findings revealed that, compared to fathers, mothers of patients had higher scores in responsibility, threat estimation, perfectionism, intolerance of uncertainty, rumination, and decreased cognitive confidence. Additionally, the mothers of patients had higher scores than the mothers of the HCs in responsibility and threat estimation, perfectionism and intolerance of uncertainty, and decreased cognitive confidence. These results suggest that these characteristics in mothers may be psychological predisposition factors contributing to the development of OCD in their children. Indeed, numerous studies support the fundamental role of these three cognitive characteristics in OCD [13,20,73,76,80,81]. These characteristics may be higher in mothers because children are more influenced by the cognitive characteristics of their primary caregiver [24,78]. It can be assumed that a cognitive pattern emphasizing heightened responsibility, threat estimation, increased vigilance for errors, and a strong need for certainty may be more easily transmitted to the child than the belief that thoughts must be controlled. In the natural flow of life, children are frequently exposed to situations that require monitoring for mistakes, making it more likely for them to internalize cognitive models of their mothers by learning. This, in turn, may contribute to the emergence of OCD-specific thought-behavior models. However, this hypothesis has not been empirically tested, and longitudinal studies investigating the complex interactions among cognitive vulnerabilities could be valuable for informing early intervention strategies. At the very least, our findings suggest that the type of parent should be taken into account in studies involving relatives, and they provide insight into the potential risk posed by the transmission of parental cognitive models in the development of OCD.

### 4.2. Cognitive Vulnerability Factors in Parents of Adolescents with Autogenous vs. Reactive Obsessions

Our second hypothesis was that parents of adolescents with autogenous obsessions would exhibit higher levels of obsessive beliefs. However, we did not find any significant differences in scale scores between the parents of adolescents with autogenous versus reactive obsessions. This result may suggest that parental factors are not strong enough to determine the type of obsession. Obsessive beliefs and metacognitions have been consistently linked to OCD; their associations with specific symptom subtypes have not been consistently demonstrated, in line with our results [19,39,53,72,75,82]. Advanced studies with larger sample sizes that allow comparisons between obsession subtypes and evaluate the modulatory effect of obsessive beliefs on neural activity may enable a more robust test of this hypothesis.

### 4.3. Relationships Between Parental Cognitive Vulnerability Factors and Clinical Scores of Patients

The study evaluated the relationships between parents’ cognitive characteristics and children’s clinical scores within the scope of the third hypothesis. Cognitive self-consciousness in mothers was associated with total disease severity, obsession, compulsion, and indecisiveness. However, no relationship was found between fathers’ cognitive self-consciousness scores and children’s clinical characteristics. No literature was found investigating the relationship between parental metacognition and OCD symptoms in children. On the other hand, a study found a significant relationship between cognitive self-consciousness and total disease severity in adults, which is similar to our findings [39]. In a recent study a reduction in OCD severity following cognitive behavioral therapy in adolescents was found to be associated with a greater reduction in cognitive self-consciousness [83]. The observed association between maternal cognitive self-consciousness and both indecisiveness and obsession scores may suggest that a tendency to continuously monitor and evaluate one’s own thought processes could influence similar tendencies in children, potentially contributing to obsessive thinking and difficulty in decision-making. In contrast, paternal scores related to responsibility, threat estimation, and perfectionism/intolerance of uncertainty were associated with children’s indecisiveness. While the mechanisms underlying these differential parental influences are not entirely clear, one possible explanation is that beliefs such as perceiving failure to prevent a negative outcome as morally equivalent to causing harm, or holding oneself responsible for unavoidable outcomes, may foster an excessive need for certainty in children. This may, in turn, increase the tendency to seek additional evidence and lead to indecision when aiming to make the “correct” choice. Feelings of guilt associated with perceived responsibility for negative outcomes may also play a role in shaping the decision-making process. Supporting this view, previous research has identified an association between OCD-related indecision and fear of guilt [84]. Indecisiveness in OCD is believed to stem from perfectionism, overestimating threats, and expecting complete certainty [85].

The negative correlation between maternal cognitive self-consciousness and poor insight in children may reflect a potential influence of maternal self-monitoring tendencies on the development of greater insight in their children. Additionally, maternal negative beliefs about worry were found to be associated with increased avoidance behaviors in children. Given that avoidance is often a strategy aimed at reducing exposure to distressing thoughts and emotions [86], it is plausible that maternal cognitions related to worry may play a role in shaping children’s avoidance tendencies through learning and modeling processes. However, no significant correlations were observed between paternal scores on any of the measured cognitive domains and children’s levels of insight, avoidance, or symptom severity, including obsession, compulsion, and total symptom scores.

A decrease in cognitive confidence is thought to be associated with pathological doubt in OCD [87]. In our study, lower cognitive confidence in fathers and higher levels of rumination in mothers were associated with increased doubt in children. However, regression analysis revealed that this relationship in fathers was explained by the need to control thoughts. Another study supporting our findings found that the need to control thoughts was associated with doubt in patients diagnosed with OCD [39]. On the other hand, it should be noted that it is not possible to infer causality and direct effects when interpreting all correlational findings. Parental cognitive characteristics may be indirectly related to clinical symptoms due to the relationship between certain cognitive characteristics transmitted to children. This situation may be particularly important when interpreting findings related to doubt. Ruminative thinking and the need to control thoughts are clearly the result of doubt. In this context, future studies with similar designs are recommended to evaluate children’s obsessive and metacognitive cognitions.

Increased responsibility and threat estimation in fathers and perfectionism and intolerance of uncertainty in mothers were found to be associated with obsessive slowness in children. No studies investigating the relationship between obsessive slowness and metacognition or obsessive cognition were found in the literature. However, our findings suggest that increased responsibility, perfectionism, and threat anticipation may lead to increased self-monitoring, which may cause obsessive slowness. Our theoretical prediction is supported by the finding of a relationship between indecision and similar parental cognitions, as well as the high correlation between indecision and obsessive slowness in children.

One of the findings of our study is that lower maternal cognitive confidence was associated with earlier onset of the disorder, whereas higher levels of maternal rumination were related to later onset. The child’s internalization of the mother’s skeptical and insecure cognitive style and accompanying behaviors may be associated with an earlier onset of the disorder. On the contrary, rumination is a more internal, mental, and passive process [51]. Unlike the behavioral consequences of decreased cognitive confidence, rumination can create an environment that affects abstract thinking, such as an anxiety-dominated environment [88,89]. Therefore, rumination triggering OCD symptoms may require a more advanced level of cognitive development that requires the child’s ability to think abstractly. To shed light on this complex relationship, longitudinal studies are needed that examine both parents’ cognitive styles and their behavioral interactions with their children. Thus, our findings will provide valuable insights into when and which parental characteristics preventive interventions for OCD should focus on.

### 4.4. Limitations and Future Directions

The results of this study should be interpreted in light of certain limitations. First, a larger sample size could have increased the generalizability of the findings. Studies with larger samples may enable a clearer understanding of the relationship between obsessive-compulsive symptom types in children and parental belief patterns and rumination. They could also allow for the use of structural equation modeling and provide more precise interpretations regarding causality. In addition, the small sample size of our study did not allow for the inclusion of parental psychopathology in the analyses. It is recommended that future studies with larger samples include psychiatric characteristics of parents as a factor in the analyses. Second, the single-center design of the study limits the generalizability of the findings. Future multi-center studies could help control for cultural and regional effects. Third, due to the cross-sectional nature of the study, it was not possible to determine temporal relationships. Longitudinal studies comparing changes in variables over time may offer a clearer picture of these relationships. Fourth, obsessive and metacognitive beliefs in children, as well as subthreshold obsessive-compulsive symptoms in healthy controls, were not assessed. Longitudinal studies examining how the relationship between cognitive and clinical features of patients and parents changes across different developmental stages could offer a clearer understanding of the environmental factors shaping the clinical presentation. Fifth, obsessive-compulsive symptoms in parents were also not evaluated. Analyzing whether the relationship between obsessive beliefs and symptoms shows similar patterns in probands and their parents could provide deeper insight into the environmental influences along the pathway from cognitive vulnerability to symptom development. Sixth, our study did not examine in detail the factors related to parents’ demographic and individual characteristics. Considering variables such as parents’ employment status, years of education, time spent with the child, the presence of other family members assisting in childcare, parental personality traits could have provided a better understanding of the differential patterns observed for mothers and fathers in the relationship between parental cognitive characteristics and ruminative thinking tendencies and clinical symptoms in children.

Parental contributions of mothers and fathers may vary across countries and cultural contexts. Seventh limitation of the study is the assumption that the primary caregiver is the mother, based on the Turkey-specific cultural context, without directly measuring this critical variable. Considering the potential influential role of this variable on the findings, future studies should identify the primary caregiver directly using objective methods.

A further limitation of our study is the failure to account for a genetic component. The observed cognitive characteristics may not be independent environmental factors but rather phenotypic manifestations of a genetic predisposition transmitted to the child. The study design does not allow us to disentangle the effect of hereditary predisposition from the effect of parental behavior. Examining the relationships between the cognitive characteristics of children at risk and not at risk for OCD and their parents could provide a stronger test of the learning hypothesis.

## 5. Conclusions

Our study is the first to investigate obsessive beliefs in both mothers and fathers, revealing parent-specific relationships. The findings show that mothers of patients have higher levels of responsibility and threat estimation, perfectionism and intolerance of uncertainty, rumination, and reduced cognitive confidence compared to fathers. Additionally, more widespread correlations were found between mothers’ obsessive beliefs, metacognition, and rumination scores and children’s clinical symptoms compared to fathers. Different combinations of cognitive characteristics transmitted from mothers and fathers may shape various manifestations of the disease. In particular, the significant relationship between mothers’ cognitive characteristics as primary caregivers and their children’s clinical symptoms provides important clues for future large-scale studies. These findings primarily suggest that the parent with closer contact may have a greater influence, thereby indirectly supporting the notion that parental cognitive characteristics can shape their children’s clinical symptoms. Consequently, the results underscore the necessity of controlling for parent type in future studies. Findings from such studies could contribute to a clearer understanding of environmental factors in the clinical diversity of OCD and help identify targets for early intervention efforts.

## Figures and Tables

**Table 1:** Clinical Characteristics of Patients.

	OCD (*n* = 45)
Age of onset	11.36 ± 2.05
CY-BOCS-O	9.46 ± 2.66
CY-BOCS-C	10.29 ± 3.15
CY-BOCS Total	19. 76 ± 5.73
CY-BOCS Insight	1.69 ± 0.46
CY-BOCS Avoidance	1.64 ± 0.61
CY-BOCS Indecisiveness	1.76 ±0.61
CY-BOCS Responsibility	1.56 ± 0.76
CY-BOCS Slowness	1.62 ± 0.61
CY-BOCS Doubt	1.07 ± 0.65
Dominant Obsession Types, n (%)	
Contamination	20 (44.4)
Aggression	10 (22.3)
Sexual	6 (13.4)
Magical/superstitious	2 (4.4)
Somatic	2 (4.4)
Religious	5 (11.1)
Treatment type, n (%)	
Pharmacotherapy	39 (86.7)
Psychotherapy	2 (4.4)
Combination	5 (8.9)
Medication type, n (%)	
Antidepressant monotherapy ^a^	28 (62.2)
Antipsychotic augmentation ^b^	17 (37.8)
Comorbidity, n (%)	
Tic disorder	5 (11.1)
Other	0 (0)

CY-BOCS: Children’s Yale-Brown Obsessive Compulsive Scale, CY-BOCS-O: Obsession subscale score of the CY-BOCS, CY-BOCS-C: Compulsion subscale score of the CY-BOCS ^a^ Fluoxetine (*n* = 31), sertraline (*n* = 14). ^b^ All augmentations were with aripiprazole.

**Table 2:** Descriptive Statistics of Parent Groups.

	OCD		Healthy Controls	
	Mother	Father	Mother	Father
Age	44.49 ± 5.99	47.96 ± 4.77	39.4 ± 7.59	42.58 ± 5.97
Psychiatric disease ^a^				
Yes	14 (31.1)	3 (6.7)	3 (6.7)	3 (6.7)
No	31 (68.9)	42 (92.3)	42 (92.3)	42 (92.3)
Psychiatric diagnoses ^a^				
OCD	11 (24.4)	0 (0)	1 (2.2)	0 (0)
MDD	3 (6.7)	3 (6.7)	1 (2.2)	1 (2.2)
GAD	0 (0)	0 (0)	1 (2.2)	1 (2.2)
Smoke ^a^				
Yes	22 (48.9)	30 (66.7)	30 (66.7)	24 (53.3)
No	23 (51.1)	15 (33.3)	15 (33.3)	21 (46.7)
OBQ-RTE	64.53 ± 23.44	49.18 ± 22.67	50.64 ± 21.61	56.87 ± 22.03
OBQ-PIU	67.64 ± 18.80	55.47 ± 20.74	57.36 ± 22.12	57.11 ± 20.33
OBQ-ICT	41.29 ± 16.72	34.62 ± 12.28	33.63 ± 14.35	35.76 ± 15.33
RTS-Q	84.60 ± 25.66	63.02 ± 21.99	70.91 ± 28.64	68.45 ± 21.82
MCQ-PW	12.33 ± 3.83	10.58 ± 4.14	11.18 ± 4.67	11.64 ± 4.18
MCQ-NW	15.31 ± 4.60	12.98 ± 4.74	12.84 ± 4.70	12.04 ± 3.37
MCQ-CC	14.96 ± 4.57	13.87 ± 10.00	10.20 ± 4.65	12.11 ± 5.10
MCQ-NCT	14.38 ± 3.56	13.78 ± 4.20	12.51 ± 3.94	13.56 ± 3.52
MCQ-CSC	18.33 ± 2.83	15.42 ± 4.52	16.11 ± 4.37	14.98 ± 3.95
PHQ-9	8.71 ± 5.5	6.24 ± 4.77	7.11± 5.79	6.71 ± 4.04

OCD: Obsessive–compulsive disorder, OBQ-RTE: Responsibility/Threat Estimation Subscale of the Obsessive Beliefs Questionnaire, OBQ-PIU: Perfectionism/Intolerance of Uncertainty Subscale of the OBQ, OBQ-ICT: Importance of Thoughts/Control of Thoughts Subscale of the OBQ, RTS-Q: Ruminative Thought Style Questionnaire, MCQ-PW: Positive Beliefs About Worry Subscale of the Metacognitions Questionnaire, MCQ-NW: Negative Beliefs About Worry Subscale of the MCQ, MCQ-CC: Cognitive Confidence Subscale of the MCQ, MCQ-NCT: Need to Control Thoughts Subscale of the MCQ, MCQ-CSC: Cognitive Self-Consciousness Subscale of the MCQ, PHQ-9: Patient Health Questionnaire, OCD: Obsessive–compulsive disorder, MDD: Major depressive disorder, GAD: Generalized anxiety disorder. **^a^** Values are presented as *n* (%).

**Table 3:** Comparison of Parents of Patients and Healthy Controls.

	Estimate	SE	df	t	P_FDR_	Post Hoc
OBQ-RTE						
Group	0.117	0.04	150.07	2.896	0.013	OCD > HCs
Parent	0.058	0.031	88	1.898	0.086	-
GroupxParent	−0.186	0.044	88	−4.284	<0.001	OCD-M > OCD-F (*p* = 0.001) OCD-M > HCs-M (*p* = 0.004)
OBQ-PIU						
Group	0.091	0.036	160.74	2.506	0.03	OCD > HCs
Parent	0.001	0.03	88	0.049	0.961	-
GroupxParent	−0.102	0.043	88	−2.39	0.036	OCD-M > OCD-F (*p* = 0.001) OCD-M > HCs-M (*p* = 0.013)
OBQ-ICT						
Group	0.086	0.037	162.36	2.302	0.037	OCD > HCs
Parent	0.023	0.031	88	0.731	0.517	-
GroupxParent	−0.087	0.044	88	−1.95	0.08	-
RTS-Q						
PHQ9	0.27	0.036	150.47	7.57	<0.001	
Group	0.075	0.029	171.92	2.548	0.028	OCD > HCs
Parent	0.006	0.027	85.95	0.23	0.859	
GroupxParent	−0.092	0.039	88.21	−2.363	0.036	OCD-M > OCD-F (*p* = 0.003) OCD-M > HCs-M (*p* = 0.012)
MCQ-PW						
Group	0.05	0.032	164.34	1.573	0.151	-
Parent	0.021	0.027	88	0.762	0.51	-
GroupxParent	−0.093	0.038	88	−2.418	0.036	OCD-M > OCD-F (*p* = 0.009)
MCQ-NW						
Group	0.074	0.031	163.24	2.424	0.035	OCD > HCs
Parent	−0.018	0.026	88	−0.687	0.533	-
GroupxParent	−0.055	0.037	88	−1.514	0.166	-
MCQ-CC						
Group	0.163	0.036	174.07	4.583	<0.001	OCD > HCs
Parent	0.068	0.034	88	2.034	0.07	-
GroupxParent	−0.136	0.048	88	−2.849	0.015	OCD-M > OCD-F (*p* < 0.001) OCD-M > HCs-M (*p* = 0.009) HCs-F > HCs-M (*p* = 0.045)
MCQ-NCT						
Group	0.061	0.026	156.3	2.345	0.036	OCD > HCs
Parent	0.036	0.021	88	1.733	0.116	-
GroupxParent	−0.06	0.03	88	−2.021	0.07	-
MCQ-CSC						
Group	0.063	0.024	170.9	2.639	0.023	OCD > HCs
Parent	−0.031	0.022	88	−1.43	0.189	-
GroupxParent	−0.053	0.031	88	−1.728	0.116	-
PHQ-9						
Group	0.08	0.063	141.33	1.255	0.248	-
Parent	−0.009	0.045	88	−0.205	0.859	
GroupxParent	−0.149	0.064	88	−2.336	0.037	OCD-M > OCD-F (*p* < 0.001)

OBQ-RTE: Responsibility/Threat Estimation Subscale of the Obsessive Beliefs Questionnaire, OBQ-PIU: Perfectionism/Intolerance of Uncertainty Subscale of the OBQ, OBQ-ICT: Importance of Thoughts/Control of Thoughts Subscale of the OBQ, RTS-Q: Ruminative Thought Style Questionnaire, MCQ-PW: Positive Beliefs About Worry Subscale of the Metacognitions Questionnaire, MCQ-NW: Negative Beliefs About Worry Subscale of the MCQ, MCQ-CC: Cognitive Confidence Subscale of the MCQ, MCQ-NCT: Need to Control Thoughts Subscale of the MCQ, MCQ-CSC: Cognitive Self-Consciousness Subscale of the MCQ, PHQ-9: Patient Health Questionnaire, OCD: Obsessive–compulsive disorder, MDD: Major depressive disorder, GAD: Generalized anxiety disorder; M: Mother, F: Father, OCD-M: Mothers of OCD patients, OCD-F: Fathers of OCD patients, HCs-M: Mothers of healthy controls, HCs-F: Fathers of HCs; F: F statistics, df: Degrees of freedom.

**Table 4:** Final Regression Models for Mothers’ Scale Scores and Clinical Measures of Patients.

Age of Onset	B	95% CI	β	*t*	*p*
MCQ-CC	−5.44	[−9.326, −1.554]	−0.381	−2.825	0.007 **
RTS-Q	4.727	[0.785, 8.669]	0.326	2.42	0.02 *
CY-BOCS-O					
MCQ-CSC	0.868	[0.387, 1.35]	0.485	3.639	<0.001 ***
CY-BOCS Slowness					
OBQ-PIU	0.809	[0.231, 1.387]	0.396	2.824	0.007 **
CY-BOCS Doubt					
RTS-Q	0.448	[0.139, 0.756]	0.408	2.927	0.005 **

CY-BOCS: Children’s Yale-Brown Obsessive Compulsive Scale, CY-BOCS-O: Obsession score of the CY-BOCS, MCQ-CC: Cognitive confidence subscale of the Metacognitions Questionnaire, MCQ-CSC: Cognitive self-consciousness subscale of the MCQ, OBQ-PIU: Perfectionism/intolerance of uncertainty subscale of the Obsessive Beliefs Questionnaire, RTS-Q: Ruminative Thought Style Questionnaire. 95% CI: 95% Confidence interval. *p < 0.05, **p < 0.01, ***p < 0.001.

## Data Availability

The data presented in this study are available on request from the corresponding author due to ethical restrictions related to the inclusion of information on children and adolescents.

## Supplementary Materials

The following supporting information can be downloaded at: https://www.mdpi.com/article/10.3390/brainsci15101093/s1, Table S1: Correlations Between Clinical Scores of Patients; Table S2: Comparison of Mothers and Fathers of Patients with Autogenous and Reactive Obsessions; Table S3: Correlations Between CY-BOCS Scores of Patients and Scale Scores of Their Mothers; Table S4: Correlations Between CY-BOCS Scores of Patients and Scale Scores of Their Fathers.
