# Supplementary material for: Obsessive Beliefs, Metacognitive Beliefs, and Rumination in Parents of Adolescents with and Without Obsessive–Compulsive Disorder: A Linear Mixed-Effects Model
Source: Brain Sci. 2025 Oct 10;15(10):1093. doi: 10.3390/brainsci15101093 (PMC12563947; doi:10.3390/brainsci15101093)
Supplement: Supplementary file 1 [file brainsci-15-01093-s001.zip › brainsci-3886586-supplementary.pdf]

Table S1. Correlations Between Clinical Scores of Patients

	**AoO**	**CY-BOCS-C**	**CY-BOCS-O**	**CY-BOCS Insight**	**CY-BOCS Avoidance**	**CY-BOCS Indecisiviness**	**CY-BOCS Responsibility**	**CY-BOCS Obsessive Slowness**	**CY-BOCS Doubt**
**CY-BOCS-C**	**r = -0.293**<br>**p = 0.005****								
**CY-BOCS-O**	**r = -0.355**<br>**p = 0.001****	**r = 0.946**<br>**p < 0.001*****							
**CY-BOCS Insight**	r = -0.119<br>p = 0.263	**r = 0.813**<br>**p < 0.001*****	**r = 0.771**<br>**p < 0.001*****						
**CY-BOCS Avoidance**	r = -0.152<br>p = 0.154	**r = 0.349**<br>**p = 0.001****	r = 0.174<br>p = 0.101	**r = 0.241**<br>**p = 0.022***					
**CY-BOCS Indecisiviness**	r = -0.075<br>p = 0.485	**r = 0.792**<br>**p < 0.001*****	**r = 0.741**<br>**p < 0.001*****	**r = 0.764**<br>**p < 0.001*****	**r = 0.251**<br>**p = 0.017***				
**CY-BOCS Responsibility**	r = 0.149<br>p = 0.162	**r = 0.378**<br>**p < 0.001*****	**r = 0.386**<br>**p < 0.001*****	**r = 0.371**<br>**p < 0.001*****	r = 0.143<br>p = 0.18	**r = 0.302**<br>**p = 0.004****			
**CY-BOCS Obsessive Slowness**	r = -0.017<br>p = 0.872	**r = 0.736**<br>**p < 0.001*****	**r = 0.663**<br>**p < 0.001*****	**r = 0.847**<br>**p < 0.001*****	**r = 0.362**<br>**p < 0.001*****	**r = 0.781**<br>**p < 0.001*****	**r = 0.512**<br>**p < 0.001*****		
**CY-BOCS Doubt**	r = 0.05<br>p = 0.641	**r = 0.243**<br>**p = 0.021***	**r = 0.268**<br>**p = 0.011***	**r = 0.218**<br>**p = 0.039***	**r = -0.225**<br>**p = 0.033***	**r = 0.27**<br>**p = 0.01***	**r = 0.522**<br>**p < 0.001*****	**r = 0.404**<br>**p < 0.001*****	
**CY-BOCS Total**	**r = -0.326**<br>**p = 0.002****	**r = 0.989**<br>**p < 0.001*****	**r = 0.984**<br>**p < 0.001*****	**r = 0.805**<br>**p < 0.001*****	**r = 0.273**<br>**p = 0.009****	**r = 0.779**<br>**p < 0.001*****	**r = 0.387**<br>**p < 0.001*****	**r = 0.712**<br>**p < 0.001*****	**r = 0.258**<br>**p = 0.014***

AoO: Age of Onset, Children's Yale-Brown Obsessive Compulsive Scale, CY-BOCS-C: Compulsion score of the CY-BOCS, CY-BOCS-O: Obsession score of the CY-BOCS

*p<0.05, **p<0.01, ***p<0.001

Table S2. Comparison of Mothers and Fathers of Patients with Autogenous and Reactive Obsessions

	Estimate	SE	df	t	P_{FDR}	Post-hoc
**OBQ-RTE**						
Group	-0.12	0.045	43	-2.678	0.072	-
Parent	-0.03	0.056	80.018	-0.544	0.992	-
Group*Parent	-0.018	0.067	43	-0.261	0.992	-
**OBQ-PC**						
Group	-0.105	0.041	43	-2.58	0.072	-
Parent	-0.045	0.046	84.825	-0.982	0.85	-
Group*Parent	0.01	0.061	43	0.162	0.992	-
**OBQ-ICT**						
Group	-0.071	0.038	43	-1.855	0.273	-
Parent	0.008	0.052	75.064	0.161	0.992	-
Group*Parent	0.017	0.057	43	0.288	0.992	-
**RTS-Q**						
Group	-0.003	0.037	72.695	-0.076	0.992	
Parent	-0.094	0.028	45.485	-3.416	0.021*	M > F
Group*Parent	0	0.04	42.396	-0.01	0.992	-
**MCQ-PW**						
Group	-0.046	0.034	43	-1.38	0.602	-
Parent	-0.002	0.044	76.617	-0.04	0.992	-
Group*Parent	-0.058	0.05	43	-1.153	0.781	-
**MCQ-NW**						
Group	-0.058	0.027	43	-2.165	0.159	-
Parent	0.018	0.047	61.541	0.382	0.992	-
Group*Parent	-0.034	0.04	43	-0.857	0.945	-
**MCQ-CC**						
Group	-0.03	0.051	86	-0.601	0.992	-
Parent	0.038	0.054	86	0.715	0.989	-
Group*Parent	-0.083	0.076	86	-1.094	0.781	-
**MCQ-NCT**						
Group	-0.017	0.029	43	-0.582	0.992	-
Parent	-0.009	0.038	77.526	-0.235	0.992	-
Group*Parent	-0.015	0.044	43	-0.34	0.992	-
**MCQ-CSC**						
Group	-0.075	0.029	43	-2.565	0.072	-
Parent	-0.013	0.031	85.962	-0.404	0.992	-
Group*Parent	-0.021	0.044	43	-0.491	0.992	-
**PHQ-9**						
Group	-0.156	0.06	43	-2.593	0.072	-
Parent	-0.07	0.099	64.433	-0.713	0.989	-
Group*Parent	-0.004	0.09	43	-0.04	0.992	-

OBQ-RTE: Responsibility/Threat estimation subscale of Obsessive Beliefs Questionnaire, OBQ-PIU: Perfectionism/intolerance of uncertainty subscale of OBQ, OBQ-ICT: Importance of thoughts/control of thoughts subscale of OBQ, RTS-Q: Ruminative Thought Style Questionnaire, MCQ-PW: Positive beliefs about worry subcale of Metacognitions Questionnaire, MCQ-NW: Negative beliefs about worry subscale of MCQ, MCQ-CC: Cognitive confidence subscale of MCQ, MCQ-NCT: Need to control

thoughts subscale of MCQ, MCQ-CSC: Cognitive self-consciousness subscale of MCQ, PHQ-9: Patient Health Questtinnaire

M: Mother, F: Father

SE: Standard error, df: Degrees of freedom, t: t statistics, p_{FDR} : FDR-corrected p value, ηp2 : Partial eta-squated,

*p<0.05, **p<0.001

Table S3. Correlations Between CY-BOCS Scores of Patients and Scale Scores of Their Mothers

	AoO	CY-BOCS-C	CY-BOCS-O	CY-BOCS-Total	CY-BOCS Insight	CY-BOCS Avoidance	CY-BOCS Indecisiviness	CY-BOCS Responsibility	CY-BOCS Obsessive Slowness	CY-BOCS Doubt
**OBQ-RTE**	r = 0.129<br>p = 0.397	r = 0.067<br>p = 0.662	r = 0.211<br>p = 0.164	r = 0.135<br>p = 0.377	r = 0.178<br>p = 0.241	r = 0.079<br>p = 0.606	r = 0.008<br>p = 0.961	r = 0.064<br>p = 0.675	r = 0.275<br>p = 0.068	**r = 0.323**<br>**p = 0.03**
**OBQ-PIU**	r = 0.199<br>p = 0.19	r = 0.222<br>p = 0.142	**r = 0.356**<br>**p = 0.016**	r = 0.287<br>p = 0.056	r = 0.267<br>p = 0.076	r = 0.088<br>p = 0.567	r = 0.034<br>p = 0.822	r = 0.169<br>p = 0.267	**r = 0.3**<br>**p = 0.046**	r = 0.246<br>p = 0.104
**OBQ-ICT**	r = 0.124<br>p = 0.415	r = 0.013<br>p = 0.93	r = 0.121<br>p = 0.429	r = 0.049<br>p = 0.75	r = 0.148<br>p = 0.333	r = 0.022<br>p = 0.886	r = 0.076<br>p = 0.62	r = 0.095<br>p = 0.533	r = 0.19<br>p = 0.212	r = 0.133<br>p = 0.385
**RTS-Q**	**r = 0.305**<br>**p = 0.041**	r = 0.034<br>p = 0.823	r = 0.116<br>p = 0.449	r = 0.073<br>p = 0.636	r = 0.089<br>p = 0.562	r = 0.056<br>p = 0.714	r = 0.005<br>p = 0.973	r = 0.037<br>p = 0.81	r = 0.133<br>p = 0.384	**r = 0.411**<br>**p = 0.005**
**MCQ-PW**	r = 0.09<br>p = 0.557	r = 0.144<br>p = 0.347	r = 0.144<br>p = 0.345	r = 0.146<br>p = 0.339	r = 0.211<br>p = 0.163	r = 0.181<br>p = 0.234	r = 0.13<br>p = 0.394	r = 0.118<br>p = 0.442	r = 0.25<br>p = 0.098	**r = 0.338**<br>**p = 0.023**
**MCQ-NW**	r = 0.194<br>p = 0.203	r = 0.036<br>p = 0.815	r = 0.116<br>p = 0.448	r = 0.034<br>p = 0.823	r = 0.062<br>p = 0.684	**r = 0.3**<br>**p = 0.045**	r = 0.038<br>p = 0.806	r = 0.087<br>p = 0.569	r = 0.02<br>p = 0.896	**r = 0.419**<br>**p = 0.004**
**MCQ-CC**	**r = -0.363**<br>**p = 0.014**	r = 0.225<br>p = 0.138	r = 0.259<br>p = 0.086	r = 0.244<br>p = 0.107	r = 0.092<br>p = 0.546	r = 0.071<br>p = 0.645	r = 0.054<br>p = 0.723	r = 0.206<br>p = 0.174	r = 0.086<br>p = 0.576	r = 0.105<br>p = 0.491
**MCQ-NCT**	r = 0.053<br>p = 0.731	r = 0.094<br>p = 0.537	r = 0.004<br>p = 0.977	r = 0.054<br>p = 0.725	r = 0.094<br>p = 0.538	r = 0.015<br>p = 0.923	r = 0.109<br>p = 0.475	r = 0.083<br>p = 0.586	r = 0.02<br>p = 0.898	r = 0.16<br>p = 0.293
**MCQ-CSC**	r = 0.053<br>p = 0.728	**r = 0.377**<br>**p = 0.011**	**r = 0.481**<br>**p = 0.001**	**r = 0.431**<br>**p = 0.003**	**r = - 0.42**<br>**p = 0.004**	r = 0.054<br>p = 0.725	**r = 0.299**<br>**p = 0.046**	r = 0.172<br>p = 0.259	**r = 0.435**<br>**p = 0.003**	r = 0.194<br>p = 0.202
**PHQ-9**	r = 0.16<br>p = 0.295	r = 0.17<br>p = 0.263	r = 0.169<br>p = 0.268	r = 0.172<br>p = 0.258	r = 0.251<br>p = 0.097	r = 0.063<br>p = 0.679	r = 0.128<br>p = 0.404	r = 0.161<br>p = 0.292	r = 0.116<br>p = 0.447	r = 0.252<br>p = 0.095

AoO: Age of Onset, Children's Yale-Brown Obsessive Compulsive Scale, CY-BOCS-C: Compulsion score of the CY-BOCS, CY-BOCS-O: Obsession score of the CY-BOCS, OBQ-RTE: Responsibility/Threat estimation subscale of Obsessive Beliefs Questionnaire, OBQ-PIU: Perfectionism/intolerance of uncertainty subscale of OBQ, OBQ-ICT: Importance of thoughts/control of thoughts subscale of OBQ, RTS-Q: Ruminative Thought Style Questionnaire, MCQ-PW: Positive beliefs about worry subcale of Metacognitions Questionnaire, MCQ-NW: Negative beliefs about worry subscale of MCQ, MCQ-CC: Cognitive confidence subscale of MCQ, MCQ-NCT: Need to control thoughts subscale of MCQ, MCQ-CSC: Cognitive self-consciousness subscale of MCQ, PHQ-9: Patient Health Questionnaire

*p<0.05, **p<0.01, ***p<0.001

Table S4. Correlations Between CY-BOCS Scores of Patients and Scale Scores of Their Fathers

	AoO	CY-BOCS-C	CY-BOCS-O	CY-BOCS-Total	CY-BOCS Insight	CY-BOCS Avoidance	CY-BOCS Indecisiviness	CY-BOCS Responsibility	CY-BOCS Obsessive Slowness	CY-BOCS Doubt
**OBQ-RTE**	r = 0.244<br>p = 0.106	r = 0.185<br>p = 0.223	r = 0.122<br>p = 0.426	r = 0.158<br>p = 0.299	r = 0.244<br>p = 0.106	r = 0.182<br>p = 0.232	**r = 0.325**<br>**p = 0.029**	**r = 0.334**<br>**p = 0.025**	**r = 0.299**<br>**p = 0.046**	r = 0.057<br>p = 0.711
**OBQ-PIU**	r = 0.155<br>p = 0.31	r = 0.191<br>p = 0.209	r = 0.138<br>p = 0.367	r = 0.169<br>p = 0.267	r = 0.287<br>p = 0.056	r = 0.227<br>p = 0.134	**r = 0.3**<br>**p = 0.045**	r = 0.156<br>p = 0.305	r = 0.276<br>p = 0.067	r = 0.147<br>p = 0.334
**OBQ-ICT**	r = 0.128<br>p = 0.403	r = 0.089<br>p = 0.563	r = 0.011<br>p = 0.944	r = 0.054<br>p = 0.726	r = 0.114<br>p = 0.456	r = 0.035<br>p = 0.82	r = 0.089<br>p = 0.561	r = 0.263<br>p = 0.081	r = 0.12<br>p = 0.432	r = 0.051<br>p = 0.741
**RTS-Q**	r = 0.008<br>p = 0.959	r = 0.182<br>p = 0.231	r = 0.176<br>p = 0.247	r = 0.182<br>p = 0.232	r = 0.058<br>p = 0.703	r = 0.2<br>p = 0.187	r = 0.293<br>p = 0.051	r = 0.178<br>p = 0.241	r = 0.046<br>p = 0.766	r = 0.177<br>p = 0.244
**MCQ-PW**	r = 0.052<br>p = 0.735	r = 0.011<br>p = 0.94	r = 0.044<br>p = 0.773	r = 0.027<br>p = 0.861	r = 0.046<br>p = 0.762	r = 0.036<br>p = 0.813	r = 0.156<br>p = 0.305	r = 0.024<br>p = 0.876	r = 0.092<br>p = 0.548	r = 0.336<br>p = 0.024
**MCQ-NW**	r = 0.227<br>p = 0.134	r = 0.039<br>p = 0.8	r = 0.134<br>p = 0.38	r = 0.084<br>p = 0.585	r = 0.022<br>p = 0.884	r = 0.066<br>p = 0.668	r = 0.099<br>p = 0.516	r = 0.093<br>p = 0.543	r = 0.087<br>p = 0.571	r = 0.288<br>p = 0.055
**MCQ-CC**	r = 0.042<br>p = 0.786	r = 0.114<br>p = 0.456	r = 0.056<br>p = 0.714	r = 0.089<br>p = 0.562	r = 0.037<br>p = 0.809	r = 0.146<br>p = 0.338	r = 0.19<br>p = 0.21	r = 0.235<br>p = 0.12	r = 0.083<br>p = 0.588	**r = 0.366**<br>**p = 0.014**
**MCQ-NCT**	r = 0.154<br>p = 0.311	r = 0.026<br>p = 0.864	r = 0.117<br>p = 0.446	r = 0.069<br>p = 0.655	r = 0.005<br>p = 0.973	r = 0.197<br>p = 0.194	r = 0.122<br>p = 0.424	r = 0.011<br>p = 0.941	r = 0.118<br>p = 0.441	**r = 0.492**<br>**p = 0.001**
**MCQ-CSC**	r = 0.151<br>p = 0.323	r = 0.134<br>p = 0.381	r = 0.15<br>p = 0.325	r = 0.143<br>p = 0.348	r = - 0.092<br>p = 0.548	r = 0.04<br>p = 0.793	r = 0<br>p = 0.998	r = 0.043<br>p = 0.777	r = 0.151<br>p = 0.323	r = 0.093<br>p = 0.543
**PHQ-9**	r = 0.244<br>p = 0.106	r = 0.04<br>p = 0.795	r = 0.006<br>p = 0.971	r = 0.025<br>p = 0.873	r = 0.008<br>p = 0.956	r = 0.186<br>p = 0.221	r = 0.059<br>p = 0.7	r = 0.111<br>p = 0.466	r = 0.029<br>p = 0.848	r = 0.434<br>p = 0.003

AoO: Age of Onset, Children's Yale-Brown Obsessive Compulsive Scale, CY-BOCS-C: Compulsion score of the CY-BOCS, CY-BOCS-O: Obsession score of the CY-BOCS, OBQ-RTE: Responsibility/Threat estimation subscale of Obsessive Beliefs Questionnaire, OBQ-PIU: Perfectionism/intolerance of uncertainty subscale of OBQ, OBQ-ICT: Importance of thoughts/control of thoughts subscale of OBQ, RTS-Q: Ruminative Thought Style Questionnaire, MCQ-PW: Positive beliefs about worry subcale of Metacognitions Questionnaire, MCQ-NW: Negative beliefs about worry subscale of MCQ, MCQ-CC: Cognitive confidence subscale of MCQ, MCQ-NCT: Need to control thoughts subscale of MCQ, MCQ-CSC: Cognitive self-consciousness subscale of MCQ, PHQ-9: Patient Health Questionnaire

*p<0.05, **p<0.01, ***p<0.001
